# Mpox Prevention Self-Efficacy and Associated Factors Among Men Who Have Sex With Men in China: Large Cross-Sectional Study

**DOI:** 10.2196/68400

**Published:** 2025-02-28

**Authors:** Quyige Gao, Shangbin Liu, Muzaibaier Tuerxunjiang, Huifang Xu, Jiechen Zhang, Gang Xu, Jianyu Chen, Yong Cai, Fan Hu, Ying Wang

**Affiliations:** 1 Dermatology Department, Tongren Hospital, Shanghai Jiao Tong University School of Medicine, Shanghai, China; 2 Public Health Research Center, Tongren Hospital, Shanghai Jiao Tong University School of Medicine, 1111 Xianxia Road, Changning District, Shanghai, 200335, China, 86 021-52039999; 3 School of Public Health, Shanghai Jiao Tong University School of Medicine, Shanghai, China; 4 College of Public Health, Shanghai University of Medicine and Health Sciences, Shanghai, China; 5 Institute of Community Medical Research, Tongren Hospital, Shanghai Jiao Tong University School of Medicine, Shanghai, China; 6 Hongqiao International Institute of Medicine, Tongren Hospital, Shanghai Jiao Tong University School of Medicine, Shanghai, China

**Keywords:** mpox, self-efficacy, men who have sex with men, MSM, monkeypox, cross-sectional study, mpox prevention self-efficacy scale

## Abstract

**Background:**

Self-efficacy in mpox (formerly known as monkeypox) prevention plays a pivotal role in promoting preventive behaviors by fostering a sense of control and motivation, especially among men who have sex with men (MSM), the population most affected by mpox in many countries.

**Objective:**

This study aims to assess the mpox prevention self-efficacy among MSM in China and identify factors influencing it, using a validated mpox prevention self-efficacy scale.

**Methods:**

From October 2023 to March 2024, a nationwide cross-sectional study was conducted among MSM (aged ≥18 years) across 6 geographic regions in China using a snowball sampling method. The recruited participants (effective response rate=2403/2481, 96.9%) were asked to complete an anonymous questionnaire designed based on prior knowledge of mpox and social cognitive theory. The mpox prevention self-efficacy scale was evaluated for construct validity using exploratory factor analysis and confirmatory factor analysis, and its reliability was assessed using the Cronbach α coefficient. Univariate and multivariable logistic regression analyses were used to examine the factors associated with mpox prevention self-efficacy among MSM.

**Results:**

A total of 2403 MSM participants were included, with a mean age of 29 (IQR 19‐39) years. Of these, 1228 (51.1%) were aged 25‐34 years, 1888 (78.6%) held a college degree or higher, and 2035 (84.7%) were unmarried. The median mpox prevention self-efficacy score was 23 (IQR 18‐28). Exploratory factor analysis retained 6 items of the mpox prevention self-efficacy scale. Confirmatory factor analysis confirmed a strong model fit (χ²₅=32.1, n=1225; *P*<.001; comparative fit index=0.991; root mean square error of approximation=0.067; standardized root mean square residual=0.02; goodness-of-fit index=0.992; normed fit index=0.990; incremental fit index=0.991; Tucker-Lewis index=0.974), with all indices within acceptable ranges. The scale demonstrated good internal consistency, with a Cronbach α of 0.859. The positive factors associated with mpox prevention self-efficacy were mpox-related knowledge (OR 1.107, 95% CI 1.070‐1.146), perceived risk awareness (OR 1.338, 95% CI 1.132‐1.583), and mpox risk perception (OR 1.154, 95% CI 1.066‐1.250), while the negative factor was age, with individuals aged 25 years and older exhibiting lower self-efficacy in mpox prevention (25‐34 years: OR 0.789, 95% CI 0.642‐0.970; 35‐44 years: OR 0.572, 95% CI 0.444‐0.736; 45 years and older: OR 0.569, 95% CI 0.394‐0.823).

**Conclusions:**

These findings highlight the critical role of targeted interventions to enhance mpox prevention self-efficacy, particularly through increasing knowledge, perceived risk awareness, and risk perception. Such interventions are especially important for middle-aged and older MSM, who may experience a decline in self-efficacy. Strengthening self-efficacy in these areas is essential for promoting sustained preventive behaviors, improving mental well-being, and contributing to more effective mpox prevention and control within the MSM community.

## Introduction

Mpox (formerly known as monkeypox) is a viral illness caused by the *Monkeypox virus*, typically characterized by a skin rash or mucosal lesions lasting 2‐4 weeks. Other symptoms include headache, fever, back pain, muscle aches, fatigue, and swollen lymph nodes [1]. The World Health Organization declared the mpox outbreak a Public Health Emergency of International Concern twice: in July 2022 and again in August 2024 [2,3]. As of June 30, 2024, there were 99,176 confirmed cases and 208 deaths reported across 116 countries, including 2460 cases in China [4]. The outbreak predominantly affects men who have sex with men (MSM), who account for 85.8% of cases with known sexual behavior data [4,5]. China, with the world’s largest MSM population—estimated at 8.3 million, nearly 1.8 times the size of the United States’ MSM population—faces unique public health challenges in controlling mpox transmission [6]. In response, the National Disease Control Bureau of China, in collaboration with the National Health Commission, implemented a prevention and control strategy for mpox [7]. This includes educating high-risk populations, promoting self-protection among MSM, and encouraging timely medical consultations. Although the outbreak in China has been effectively controlled since September 2023, the high mobility and risk behaviors of the MSM population require continued vigilance and proactive measures [8].

According to social cognitive theory, self-efficacy, defined as the belief in one’s ability to perform actions necessary to achieve desired outcomes [9], is a key factor in initiating and maintaining health-related behaviors [10]. Self-efficacy has been found to mediate the relationship between HIV self-testing provision and testing frequency among MSM [11], while it also directly influences the uptake of pre-exposure prophylaxis in this population [12], suggesting that enhancing self-efficacy may serve as an effective strategy to improve preventive health behaviors in at-risk populations. In the context of mpox prevention, self-efficacy is particularly critical for MSM, as it influences their confidence in adopting preventive measures, such as consistent condom use, vaccination, testing, and seeking timely medical care [13-16]. High self-efficacy has been associated with greater motivation to engage in mpox prevention behaviors and better adherence to preventive strategies, even among MSM who may continue to engage in high-risk behaviors despite awareness of mpox [17,18]. Given these findings, understanding mpox prevention self-efficacy in MSM is essential for informing targeted interventions and enhancing prevention strategies by identifying priority groups and potential barriers to mpox prevention and control.

A significant challenge in studying self-efficacy in mpox prevention is the lack of standardized measurement tools. Previous studies have primarily relied on single-item measures to assess the association between self-efficacy and mpox vaccination intentions [16,19] or used generalized instruments such as the General Self-Efficacy Scale or isolated questions [19,20]. Although these tools provide a general assessment, they fail to capture the nuanced dimensions of self-efficacy specific to mpox prevention. This limitation is particularly pronounced in the MSM population, where tailored assessments are essential to address unique behavioral and psychosocial factors. The lack of specificity and comprehensiveness in these existing measures not only undermines the validity of research findings, but also hampers the development of targeted interventions. Therefore, there is a clear need for tools specifically tailored to mpox prevention, integrating established scales with the unique characteristics of mpox.

Existing research indicates that the self-efficacy of MSM in preventing mpox may be influenced by multiple factors at various levels, including sociodemographic characteristics (eg, age, education level) [21], behavioral factors (eg, engagement in unsafe sexual practices) [22], and disease-related factors (eg, disease-related knowledge, risk perception) [23]. Research indicates that disease-related knowledge forms the basis for accurate health beliefs and enhances confidence in adopting safe sexual behaviors. A positive correlation has been observed between the level of disease-related knowledge and self-efficacy [24,25]. Additionally, mpox perceived risk awareness refers to individuals’ recognition of the potential harm or likelihood of mpox in their surroundings [26,27], while risk perception pertains to the subjective judgment of one’s personal susceptibility to mpox [28]. Evidence suggests that risk perception is a key trigger for both preventive awareness and action [29]. Along with disease-specific knowledge and awareness of environmental health risks, risk perception significantly influences mpox prevention awareness and behaviors [30]. However, the relationship between mpox perceived risk awareness, mpox risk perception, and self-efficacy, particularly among MSM, remains an underexplored area.

Given the critical role of self-efficacy in sustaining preventive motivation and goal-directed behavior among MSM, it is essential to assess it within the context of mpox prevention. While existing research on mpox prevention in MSM has primarily focused on vaccination willingness and testing behavior [15,31-33], there is a notable gap in understanding the self-efficacy of MSM in mpox prevention and the factors that influence it. Identifying these factors can help guide the development of targeted interventions. This study aims to evaluate mpox prevention self-efficacy among MSM in China, examining the impacts of sociodemographic characteristics, mpox-related knowledge, perceived risk awareness, and risk perception. By identifying these factors, the study seeks to provide a theoretical basis for designing more effective mpox prevention strategies tailored to this vulnerable population, while simultaneously contributing to improved behavioral outcomes and mental well-being within the MSM community.

## Methods

### Study Design

From October 2023 to March 2024, a nationwide cross-sectional study was conducted among MSM (age ≥18 years) across 6 geographic regions in China. Survey sites were located in the Northwest (Xinjiang Uyghur Autonomous Region), Northeast (Liaoning Province), Central (Shaanxi Province), Southwest (Yunnan Province), Southeast (Guangdong Province), and Eastern coastal region (Shanghai Municipality). Details of the program have been described previously [34].

### Participant Recruitment and Data Collection

Eligibility criteria for participants included: (1) born male; (2) aged ≥18 years old; (3) have ever engaged in sex with men within the last six months; (4) primarily reside in one of the selected locations during the same period; and (5) agree to participate in the survey. Simultaneously, participants were excluded if they: (1) completed the questionnaire within 300 seconds; (2) failed the quality control questions (eg, Is Guangzhou, Shanghai, Beijing, or Shenzhen the capital city in China?); and (3) had an IP address indicating a location outside the target regions.

Participants were recruited from 6 cities in China with the assistance of local governmental centers for disease control and non-governmental organizations (NGOs), using a snowball sampling method. The NGOs routinely engaged with the MSM population in the region and were well-acquainted with local disease prevention and control initiatives. Initially, NGO staff, serving as primary investigators in each region, underwent training on recruitment procedures. They were tasked with recruiting 5‐10 “seed” participants (MSM individuals with good outreach potential) who met the inclusion criteria from each region. These seed participants were then asked to recruit additional participants from their social networks until the target sample size was achieved. Data collection was conducted entirely anonymously through an online survey. Participants accessed the electronic questionnaire via QR code scanning at designated survey locations. NGO staff assisted participants in completing the online survey, providing technical support and non-directive guidance as necessary, in accordance with the study protocol outlined previously [34].

After thorough verification, a total of 2481 questionnaires were collected, of which 2403 were deemed valid, yielding an effective response rate of 96.9%. The collected data underwent thorough cleaning and processing, including checks for missing data, outliers, and logical inconsistencies, with necessary adjustments and corrections made accordingly.

### Ethical Considerations

The study was approved by Shanghai University of Medicine and Health Sciences (approval number 2023-MSMMPOX-22‐310222197604080237) on October 8, 2023. All participants signed electronic informed consent forms. Data collection was conducted anonymously using an electronic questionnaire administered through a secure, privacy-compliant platform (Wenjuanxing) via QR code scanning. To encourage participation, each individual who completed the survey and passed quality checks received Chinese yuan (CNY) 80 (about US $10.96). To ensure confidentiality, deidentified data were used for subsequent analysis.

### Variables

#### Sociodemographic Characteristics

A range of sociodemographic characteristics (including age, education level, marital status, and monthly income) and disease diagnoses (including hypertension, diabetes, and hyperlipidemia) were assessed via self-report.

#### Mpox Prevention Self-Efficacy

Based on Bandura’s self-efficacy theory and established scales [19], such as the HIV Prevention Self-Efficacy Scale [35] and the Self-Efficacy for HIV Prevention Behaviors Scale [36], we developed a 6-item mpox prevention self-efficacy scale tailored to the characteristics of MSM and mpox prevention. The scale aimed to assess individuals’ confidence in their ability to effectively prevent mpox. Each item was rated on a 5-point Likert scale, ranging from “1 strongly disagree” to “5 strongly agree,” as described in Table S1 in Multimedia Appendix 1. The composite variable was calculated by summing the scores of these items (range: 6‐30, Cronbach α=0.859), with a higher score indicating higher mpox prevention self-efficacy. A total score was categorized into 2 groups based on the median (median=23, skewness=−0.94): no/low self-efficacy (<23) and self-efficacy (≥23). When the independent variables are uncorrelated and exhibit a skewed distribution, the median is considered an effective tool for segmentation, which is the case for the variables in our study [37,38].

#### Mpox-Related Knowledge

Mpox-related knowledge included 12 yes/no questions designed to evaluate participants’ understanding of mpox, covering various aspects such as pathogenesis (eg, “Mpox is a viral infectious disease”), epidemiological characteristics (eg, “Mpox can be transmitted through mucous membranes and broken skin”), clinical manifestations (eg, “The only symptom of mpox is a rash”), and preventive measures (eg, “Smallpox vaccination can be used to prevent mpox”). The overall score was calculated by counting the number of correct answers, with each correct response scoring 1 point. Higher scores indicated greater knowledge of mpox, with a possible range of 0‐12 (Cronbach α=0.80).

#### Mpox Perceived Risk Awareness

Perceived risk awareness, primarily utilized in public environmental health research, refers to the level of participants’ attentiveness to potential risks in their environment [26,27]. We developed a composite variable for mpox perceived risk awareness based on 4 statements [28], each rated on a 5-point Likert scale (from “1=strongly agree” to “5=strongly disagree”). A composite scale score was calculated by summing the item scores, with higher scores indicating greater awareness of risks associated with mpox (range: 0‐20; Cronbach α=0.906). A total score was categorized into 2 groups based on the median: low perceived risk awareness (<17.00) and high perceived risk awareness (≥17.00).

#### Mpox Risk Perception

Risk perception refers to how people perceive the likelihood of experiencing harm or loss due to a disease and represents a subjective judgment of disease susceptibility [28]. It is a core construct in many health behavior theories, such as the Protection Motivation Theory and the Health Belief Model [39]. In the Health Belief Model, risk perception encompasses perceived susceptibility (belief in the likelihood of contracting a disease) and perceived severity (the perceived seriousness of the disease) [40]. In this study, considering the substantial variability in the clinical manifestations of mpox among individuals, mpox risk perception specifically focuses on participants’ beliefs regarding the likelihood of contracting mpox. The statement “I believe I am someone who is likely to contract mpox” was used to assess personal mpox risk perception on a 5-point Likert scale, ranging from “strongly disagree” to “strongly agree” [28].

### Statistical Analysis

First, descriptive statistics were used to characterize the distribution of the variables. Continuous variables, which were non-normally distributed, were presented as medians and IQRs, while categorical variables were described using frequencies and percentages. Second, the content validity of the questionnaire was evaluated using the critical ratio method and Spearman correlation coefficient (N=2403). The total scores of the mpox prevention self-efficacy scale were ranked from low to high, with the bottom 27% classified as the low-score group and the top 27% as the high-score group. The discrimination of each item was tested using an independent samples *t* test between these 2 groups. Additionally, the total sample was randomly split into 2 subsets using a random number method: one subset (n=1178) was used for exploratory factor analysis (EFA), and the other (n=1225) for confirmatory factor analysis (CFA), to cross-validate the scale. For EFA, the feasibility of factor analysis was assessed using the Kaiser-Meyer-Olkin test and Bartlett test of sphericity. For CFA, model fit was assessed using several fit indices: the chi-square goodness-of-fit (*χ²/df*), goodness-of-fit index (GFI), root mean square error of approximation (RMSEA), standardized root mean square residual (SRMR), normed fit index (NFI), comparative fit index (CFI), Tucker-Lewis index (TLI), and incremental fit index (IFI). The average variance extracted and composite reliability were also computed to assess the scale’s convergent and discriminant validity. Third, univariate logistic regression was employed to examine the crude associations between the outcome variable and the variables of interest (ie, mpox-related and demographic variables). Fourth, a multivariable logistic regression analysis was performed to identify factors associated with mpox prevention self-efficacy, using the Enter method. Odds ratios (OR) and 95% CIs were estimated. Variance inflation factors were calculated to ensure the independence of each variable in the model, as described in Table S2 in Multimedia Appendix 2. The Hosmer-Lemeshow test indicated a good model fit (*χ²_8_*=6.047, *P*=.642). Descriptive, univariate, and multivariable analyses were conducted using SPSS software (version 22.0; IBM Corp). The reliability and validity of the questionnaire were assessed using AMOS software (version 28.0; IBM Corp). Forest plots were generated using GraphPad Prism software (version 9.5; GraphPad Software Inc).

## Results

### Demographic Characteristics

The median age of participants was 29 (IQR 19‐39) years old. In the 30-point self-efficacy score, the median mpox prevention self-efficacy score was 23 (IQR 18‐28). The majority held a college degree or higher (n=1888, 78.6%), were unmarried (n=2035, 84.7%), and did not have diabetes (n=2334, 97.1%), hypertension (n=2248, 93.6%), or hyperlipidemia (n=2181, 90.8%). Among the participants, those with a college education or higher (1017/1888, 53.9%) and those who were divorced or widowed (62/113, 54.9%) demonstrated higher levels of mpox prevention self-efficacy (Table 1).

**Table 1.** Background characteristics for all included MSM^a^ in 6 Chinese cities according to mpox prevention self-efficacy (N=2403), from a survey conducted from October 2023 to March 2024.

Characteristic	Overall	No/low self-efficacy	Self-efficacy	*P* value
Age (years)				
Median (IQR)	29 (19‐39)	30 (19‐41)	28 (19‐37)	<.001
18‐24, n (%)	564 (23.5)	225 (20)	339 (26.5)	<.001
25‐34, n (%)	1228 (51.1)	563 (50.1)	665 (52)	
35‐44, n (%)	459 (19.1)	249 (22.2)	210 (16.4)	
≥45, n (%)	152 (6.3)	86 (7.7)	66 (5.2)	
Education level, n (%)				
Junior high school and below	157 (6.5)	85 (54.1)	72 (45.9)	.16
Senior high school	358 (14.9)	167 (46.7)	191 (53.4)	
College or above	1888 (78.6)	871 (46.1)	1017 (53.9)	
Marital status, n (%)				
Unmarried	2035 (84.7)	937 (46)	1098 (54)	.11
Married	255 (10.6)	135 (52.9)	120 (47.1)	
Divorced or widowed	113 (7.4)	51 (45.1)	62 (54.9)	
Income (CNY^b^), n (%)				
≤3000 (US $414)	502 (20.9)	254 (50.6)	248 (49.4)	.01
3001‐6000 (US $414-$828)	851 (35.4)	403 (47.4)	448 (52.6)	
6001‐12,000 (US $828-$1656)	802 (33.4)	340 (42.4)	462 (57.6)	
>12,000 (US $1656)	248 (10.3)	126 (50.8)	122 (49.2)	
Hypertension, n (%)				
No	2248 (93.6)	1049 (46.7)	1199 (53.3)	.80
Yes	155 (6.5)	74 (47.7)	81 (52.3)	
Diabetes, n (%)				
No	2334 (97.1)	1086 (46.5)	1248 (53.5)	.24
Yes	69 (2.9)	37 (53.6)	32 (46.4)	
Hyperlipemia, n (%)				
No	2181 (90.8)	991 (45.4)	1190 (54.6)	<.001
Yes	222 (9.2)	132 (59.5)	90 (40.5)	
Mpox-related knowledge, median (IQR)	8 (5-11)	8 (5-11)	9 (6‐12)	<.001
Perceive risk awareness, n (%)				
Low	1173 (48.8)	609 (51.9)	564 (48.1)	<.001
High	1230 (52.2)	514 (41.8)	716 (58.2)	
Mpox risk perception, median (IQR)	3 (2-4)	3 (2-4)	4 (3-5)	<.001

a MSM: men who have sex with men.

b CNY: Chinese yuan. An exchange rate of CNY 1=US $0.14 has been applied.

### Reliability and Validity Assessment of the Mpox Prevention Self-Efficacy Scale

#### Content Validity of the Scale

The results of the independent samples *t* test revealed statistically significant differences between the high-score group (top 27%) and the low-score group (bottom 27%) across all items (*P*<.001), as described in Table S1 in Multimedia Appendix 1. The critical ratio was 65.957, indicating that the scale possesses strong discriminatory power. Furthermore, Pearson correlation analysis demonstrated that the correlation coefficients between the total questionnaire score and the scores of the 6 items ranged from 0.746 to 0.817 (*P*<.001), suggesting that each item is highly representative of the overall construct.

#### Construct Validity of the Scale

The Kaiser-Meyer-Olkin measure of sampling adequacy was 0.817, and the Bartlett test yielded a χ²_15_ value of 3442.5 (*P*<.001), indicating that the data were suitable for factor analysis. EFA of the baseline data extracted a single factor with an eigenvalue greater than 1.00, accounting for 60.02% of the total variance. The factor loadings for each item on the common factor ranged from 0.630 to 0.799 (Table 2).

**Table 2.** Factor loadings of the mpox prevention self-efficacy scale for men who have sex with men (n=1225).

Path	Standardized factor loadings	SE	*P* value	CR^a^	AVE^b^
C1_1<--Self_efficacy	0.630			0.845	0.478
C1_2<--Self_efficacy	0.640	0.043	<.001		
C1_3<--Self_efficacy	0.645	0.070	<.001		
C1_4<--Self_efficacy	0.664	0.058	<.001		
C1_5<--Self_efficacy	0.752	0.056	<.001		
C1_6<--Self_efficacy	0.799	0.055	<.001		

a CR: composite reliability.

b AVE: average variance extracted.

We then conducted a CFA on the baseline data to evaluate the global model fit of the factor structure suggested by the EFA, as described in Figure S1 in Multimedia Appendix 3. The overall model fit was good (*χ²_5_*=32.1, n=1225; *P*<.001; CFI=0.991; RMSEA=0.067; SRMR=0.02; GFI=0.992; NFI=0.990; IFI=0.991; TLI =0.974). All indices were within acceptable ranges, indicating that the model was well-aligned with the data (Table 3).

**Table 3.** Model fit index of the mpox prevention self-efficacy scale for men who have sex with men (n=1225).

Model fitting index	Chi-square (*df*)^a^	RMSEA^b^	SRMR^c^	GFI^d^	CFI^e^	NFI^f^	TLI^g^	IFI^h^
Reference value	<3.000	<0.080	<0.050	>0.900	>0.900	>0.900	>0.900	>0.900
Model value	6.416	0.067	0.0184	0.992	0.991	0.990	0.974	0.991

a Chi-square (*df*): *χ* goodness-of-fit.

b RMSEA: root mean square error of approximation.

c SRMR: standardized root mean square residual.

d GFI: goodness-of-fit index.

e CFI: comparative fit index.

f NFI: normed fit index.

g TLI: Tucker-Lewis index.

h IFI: incremental fit index.

#### Reliability Analysis of the Scale

The Cronbach α coefficient for the mpox prevention self-efficacy scale was 0.859, indicating good internal consistency (Cronbach α>0.80).

### Factors Associated With Mpox Prevention Self-Efficacy

#### Univariate Logistic Regression Analysis

Univariate analysis revealed that higher levels of mpox-related knowledge, greater perceived risk awareness, and increased mpox risk perception were significantly associated with higher mpox prevention self-efficacy. Conversely, older MSM demonstrated lower self-efficacy in mpox prevention. All these differences were statistically significant (Table 4).

**Table 4.** Univariate logistic regression analysis of mpox prevention self-efficacy among men who have sex with men in 6 Chinese cities (N＝2403), from a survey conducted from October 2023 to March 2024.

Characteristic	Odds ratio	95% CI	*P* value
Age group, years (reference: 18‐24)			
25‐34	0.784	0.640‐0.960	.02
35‐44	0.560	0.436‐0.718	<.001
≥45	0.509	0.355‐0.732	<.001
Education level (reference: junior high school and below)			
Senior high school	1.350	0.927‐1.967	.12
College or above	1.378	0.994‐1.911	.05
Marital status (reference: unmarried)			
Married	0.759	0.584‐0.985	.04
Divorced or widowed	1.037	0.709‐1.518	.85
Income, CNY^a^ (reference: ≤3000 [US $414])			
3001‐6000 (US $414-$828)	1.139	0.913‐1.420	.25
6001‐12,000 (US $828-$1656)	1.392	1.112‐1.741	.004
>12,000 (US $1656)	0.992	0.732‐1.344	.96
Disease-related factor			
Mpox-related knowledge	1.124	1.088‐1.163	<.001
Perceived risk awareness (high versus low)	1.338	1.132‐1.583	.001
Mpox risk perception	1.157	1.070‐1.252	<.001

a CNY: Chinese yuan. The exchange rate used is 1 CNY =0.14USD.

#### Multivariable Logistic Regression Analysis

**Figure 1.**
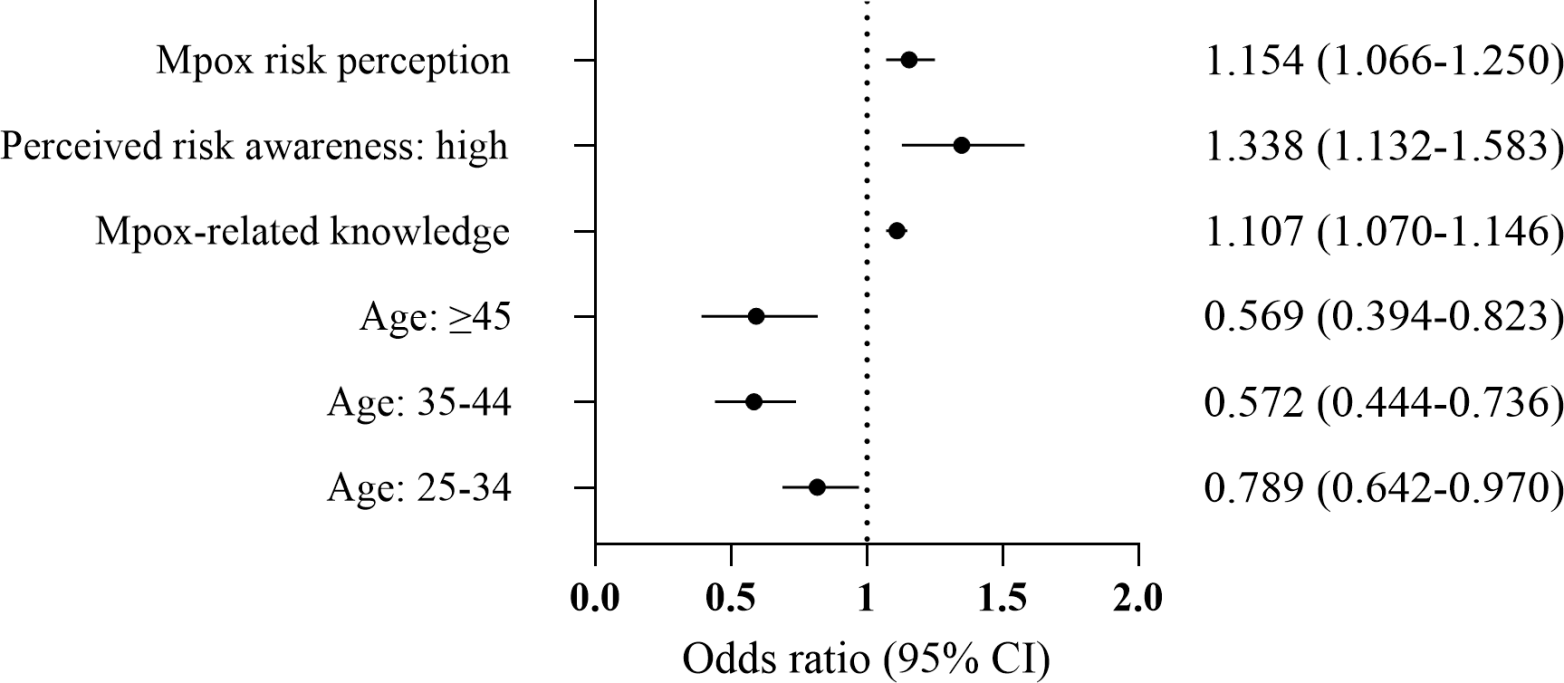
Multivariable logistic regression analysis of mpox prevention self-efficacy among MSM in 6 Chinese cities (N=2403), from a survey conducted from October 2023 to March 2024. The categories “18‐24 years old” and “low perceived risk awareness” were used as references in the model. MSM: men who have sex with men.

Variables with statistically significant differences from the univariate analysis were included in the multivariable logistic regression model (Figure 1). In this analysis (Y: mpox prevention self-efficacy, 0=no/low self-efficacy, 1=self-efficacy), the positive factors associated with mpox prevention self-efficacy were mpox-related knowledge (OR 1.107, 95% CI 1.070‐1.146), perceived risk awareness (OR 1.338, 95% CI 1.132‐1.583), and mpox risk perception (OR 1.154, 95% CI 1.066‐1.250). Individuals over the age of 25 years may exhibit lower self-efficacy in mpox prevention (25‐34 years: OR 0.789, 95% CI 0.642‐0.970; 35‐44 years: OR 0.572, 95% CI 0.444‐0.736; 45 years and older: OR 0.569, 95% CI 0.394‐0.823).

## Discussion

### Principal Findings

This study represents one of the earliest investigations into mpox prevention self-efficacy and its influencing factors among MSM. Given that the mpox outbreak was once again declared a Public Health Emergency of International Concern in 2024, concerns about the potential spread of the virus—particularly within the MSM community—have intensified. This highlights the critical need for effective preventive strategies tailored to this high-risk group. Considering the crucial role of self-efficacy in health behaviors and disease prevention, our findings offer valuable public health insights for managing mpox outbreaks and enhancing the psychological and behavioral well-being of the MSM population.

### Comparison With Prior Work

The study found that the median mpox prevention self-efficacy score was 23 (IQR 18‐28), with a negatively skewed distribution of self-efficacy scores. This suggests that most participants demonstrated relatively high self-efficacy scores in preventing mpox, though a small proportion reported lower self-efficacy levels. These results indicate that, while the MSM community generally exhibits strong preparedness for mpox prevention, targeted interventions may be necessary for individuals with lower self-efficacy. Establishing and reinforcing high levels of mpox prevention self-efficacy is essential, as it plays a critical role in identifying at-risk populations and implementing targeted interventions [41,42]. Strengthening self-efficacy, particularly among those with lower initial scores, can improve both individual and community-level responses to prevention efforts, thereby contributing to more effective mpox control strategies. Therefore, public health programs should prioritize efforts to improve self-efficacy, focusing on education and risk awareness to increase confidence in preventive actions.

The study found that MSM with an education level of junior high school or below exhibited lower levels of self-efficacy. These findings are consistent with the demographic characteristics observed in previous studies of MSM with mpox [43,44]. Higher education levels may be associated with increased awareness of self-protection measures, which may contribute to higher self-efficacy and a reduction in risky behaviors [45]. This underscores the potential effectiveness of educational interventions aimed at enhancing self-efficacy, particularly for individuals with lower education levels.

An interesting finding of this study is that the proportion of married MSM exhibiting high self-efficacy in mpox prevention was lower compared to their unmarried, divorced, or widowed counterparts, despite the lack of statistical significance (*P*>.05). This trend may be explained by the fact that unmarried, divorced, or widowed MSM tend to receive less familial support, which may drive them to be more proactive in seeking preventive and self-protective measures to avoid infection [46]. Previous studies have also shown that divorced or widowed MSM tend to have higher levels of mpox-related knowledge compared to married individuals [47], possibly because the latter may be less inclined to seek out information or self-protective behaviors related to mpox [48]. This observation warrants further exploration, as it may suggest that marital status could still influence self-efficacy in mpox prevention, albeit not significantly in this study.

Accurate measurement of self-efficacy is fundamental for developing effective mpox prevention strategies [49]. In this study, the mpox prevention self-efficacy scale was validated and demonstrated strong reliability, validity, and practical utility. Although the General Self-Efficacy Scale, originally developed by Schwarzer and Jerusalem [50], is widely used due to its excellent internal consistency and test-retest reliability, it lacks the specificity required for assessing self-efficacy in the MSM population and in the context of mpox prevention, limiting its applicability in this context. Previous research on mpox predominantly has largely relied on isolated questions to assess self-efficacy, without employing a comprehensive or systematic scale tailored to the MSM community [51]. To fill this gap, this study designed and evaluated the mpox prevention self-efficacy scale specifically tailored for MSM. The scale offers a more precise tool for assessing self-efficacy and its related factors among MSM, providing valuable insights for future research and interventions aimed at improving mpox prevention strategies.

Univariate analysis revealed a significant negative association between age and mpox prevention self-efficacy, a relationship that remained significant in the multivariable regression model. These findings indicate that older MSM may exhibit lower levels of self-efficacy in mpox prevention compared to their younger counterparts (aged 18‐24 years). This association may be partially attributed to older MSM having reduced access to mpox-related information. Age has been identified as a key determinant of mpox-related knowledge [52], with older individuals potentially having less exposure to relevant health information, which may contribute to lower awareness, confidence, and self-efficacy in preventing mpox. In contrast, younger MSM, being more familiar with using the internet, are more likely to access mpox-related information. Furthermore, previous studies have shown that older MSM are less willing to change their sexual behavior compared to their younger counterparts [37]. Specifically, older MSM are more likely to maintain risky sexual behaviors even after becoming aware of the health risks associated with mpox, highlighting the need for tailored interventions aimed at this population to enhance their self-efficacy in preventing mpox. Therefore, age-specific communication strategies should be considered in mpox prevention efforts, particularly targeting middle-aged and older MSM through effective media channels. Given that MSM extensively use the internet for socializing, seeking sexual partners, and accessing sexual health information [53,54], eHealth technologies have emerged as promising tools for delivering prevention interventions to this population [55]. Research has shown that enhanced behavioral interventions, such as tailored strategies, can lead to reductions in unprotected sex, while advanced eHealth technologies, including social media platforms, are associated with increased health screening in the MSM community [56]. Therefore, eHealth-based interventions, incorporating targeted communication strategies and educational tools, can be effectively implemented on widely used social platforms or websites to enhance mpox prevention self-efficacy, particularly in populations with lower levels of self-efficacy. Tailored interventions focusing on improving self-efficacy among older and less educated MSM may be particularly beneficial, as these groups tend to exhibit lower self-efficacy, which may increase their susceptibility to infection. These findings underscore the importance of demographic factors in informing targeted mpox prevention strategies, especially in guiding surveillance and health education practices.

This study found that mpox-related knowledge, perceived risk awareness, and risk perception were positively correlated with self-efficacy in mpox prevention. Previous research has demonstrated that attitudes towards a disease are closely linked to disease-related knowledge, suggesting that lower levels of knowledge among MSM are associated with more negative attitudes and decreased intentions to engage in mpox prevention behaviors [57]. Mpox perceived risk awareness reflects beliefs about the potential harm or likelihood of mpox in their surroundings, while risk perception represents a subjective judgment of personal susceptibility to the disease. According to social cognitive theory, individuals who recognize the severity of a negative health condition, believe that it can be avoided, maintain a positive attitude toward recommended actions, and have confidence in their ability to successfully execute these actions are more likely to engage in health-related behaviors [22,58]. Therefore, mpox prevention efforts among MSM should include further public health education and training to enhance perceived risk awareness, provide targeted risk communication, and ensure the privacy and confidentiality of services, which are crucial for effective mpox prevention within this population.

This research has several strengths. First, as one of the earliest studies in China examining mpox prevention self-efficacy among MSM, the findings carry significant public health implications for the prevention and control of mpox outbreaks in the country, while also enhancing the psychological and behavioral well-being of the MSM community. Furthermore, the study’s multicenter design contributes to the generalizability of the results, as it incorporates a diverse sample from various regions across China.

### Limitations

Despite the valuable insights provided by the study, several limitations must be acknowledged. First, participant recruitment was facilitated by government centers for disease control departments and NGOs using a snowball sampling method, which was used to improve the efficiency of identifying and recruiting members of the hidden MSM population [59]. Although this approach is effective for accessing hard-to-reach groups, it inherently introduces selection bias, as the sample composition is influenced by the characteristics of initial recruits (seeds) and the subsequent limited recruitment chains. Consequently, this may restrict the generalizability of our findings. Second, the sensitive nature of some inquiries, combined with self-reported data, may lead to information bias. Third, the cross-sectional design restricts the ability to infer causality, highlighting the need for future longitudinal studies to further explore the temporal relationships between mpox-related knowledge, perceived risk awareness, risk perception, and self-efficacy. Such prospective research could also evaluate the impact of targeted interventions aimed at enhancing self-efficacy in mpox prevention among MSM.

### Conclusion

This study examined the mpox prevention self-efficacy of MSM and its influencing factors, based on the design and evaluation of the mpox prevention self-efficacy scale. The findings demonstrated significant associations between mpox prevention self-efficacy and sociodemographic characteristics, as well as mpox-related factors. These results underscore the importance of targeted interventions to enhance mpox prevention self-efficacy, particularly through efforts to increase mpox-related knowledge, perceived risk awareness, and risk perception. Special attention should be given to middle-aged and older MSM, who may experience a decline in self-efficacy. Future research should adopt longitudinal designs to better understand the temporal relationships between self-efficacy and related factors, thereby providing more robust evidence to guide the development of effective mpox prevention strategies. Ultimately, these efforts are crucial for improving mpox prevention outcomes and promoting the overall health of the MSM community.

## Supplementary material

10.2196/68400 Multimedia Appendix 1 Independent samples *t* test discrimination results of the mpox prevention self-efficacy scale (N=2403).

10.2196/68400 Multimedia Appendix 2 Results of collinearity diagnosis for the multivariable logistic regression analysis of mpox prevention self-efficacy among MSM in 6 Chinese cities (N=2403), from a survey conducted from October 2023 to March 2024.

10.2196/68400 Multimedia Appendix 3 Standardized one-factor structural model of the mpox prevention self-efficacy scale (n=1225).

## Abbreviations

CFA: confirmatory factor analysis
CFI: comparative fit index
EFA: exploratory factor analysis
GFI: goodness-of-fit index
IFI: incremental fit index
MSM: men who have sex with men
NFI: normed fit index
NGO: nongovernmental organization
OR: odds ratio
RMSEA: root mean square error of approximation
SRMR: standardized root mean square residual
TLI: Tucker-Lewis index
