# Supplementary material for: Mpox Prevention Self-Efficacy and Associated Factors Among Men Who Have Sex With Men in China: Large Cross-Sectional Study
Source: JMIR Public Health Surveill. 2025 Feb 28;11:e68400. doi: 10.2196/68400 (PMC11887935; doi:10.2196/68400)
Supplement: Multimedia Appendix 1 [file publichealth-v11-e68400-s001.doc]

**Table S1. Independent samples *t* test discrimination results of the mpox prevention self-efficacy scale (N＝2403).**

| **Items** | ***t* value** | **Cronbach α** |
| --- | --- | --- |
| C1. I am confident in correctly using protective equipment, such as condoms, to reduce the risk of mpox infection. | 34.90^***^ | 0.86 |
| C2. I believe I can protect myself from mpox infection. | 37.67^***^ |
| C3. I can actively obtain health information about mpox from my family. | 45.41^***^ |
| C4. I can actively obtain health information about mpox from my friends. | 46.85^***^ |
| C5. I can actively participate in community or media-based mpox prevention and health promotion activities. | 40.47^***^ |
| C6. I can follow the health guidance provided by professionals (doctors/CDC personnel) for mpox prevention. | 31.05^***^ |
