# Supplementary material for: Mpox Prevention Self-Efficacy and Associated Factors Among Men Who Have Sex With Men in China: Large Cross-Sectional Study
Source: JMIR Public Health Surveill. 2025 Feb 28;11:e68400. doi: 10.2196/68400 (PMC11887935; doi:10.2196/68400)
Supplement: Multimedia Appendix 2 [file publichealth-v11-e68400-s002.doc]

**Table S2. Results of collinearity diagnosis** **for the multivariable logistic regression analysis of mpox prevention self-efficacy among MSM in 6 Chinese cities (N＝2403), from a survey conducted from October 2023 to March 2024.**

| **Characteristic** | **VIF^a^** | **Tolerance** |
| --- | --- | --- |
| **Demographics** | | |
| Age group (reference: 18-24) |  |  |
| 25-34 | 1.572 | 0.636 |
| 35-44 | 1.487 | 0.673 |
| ≧45 | 1.209 | 0.827 |
| **Disease-related factor** | | |
| Mpox-related knowledge | 1.053 | 0.950 |
| Perceived risk awareness (high versus low) | 1.062 | 0.941 |
| Mpox risk perception | 1.012 | 0.988 |

**^a^**VIF: Variance Inflation Factor.
