# Supplementary figures and images for: Mpox Prevention Self-Efficacy and Associated Factors Among Men Who Have Sex With Men in China: Large Cross-Sectional Study
Source: JMIR Public Health Surveill. 2025 Feb 28;11:e68400. doi: 10.2196/68400 (PMC11887935; doi:10.2196/68400)

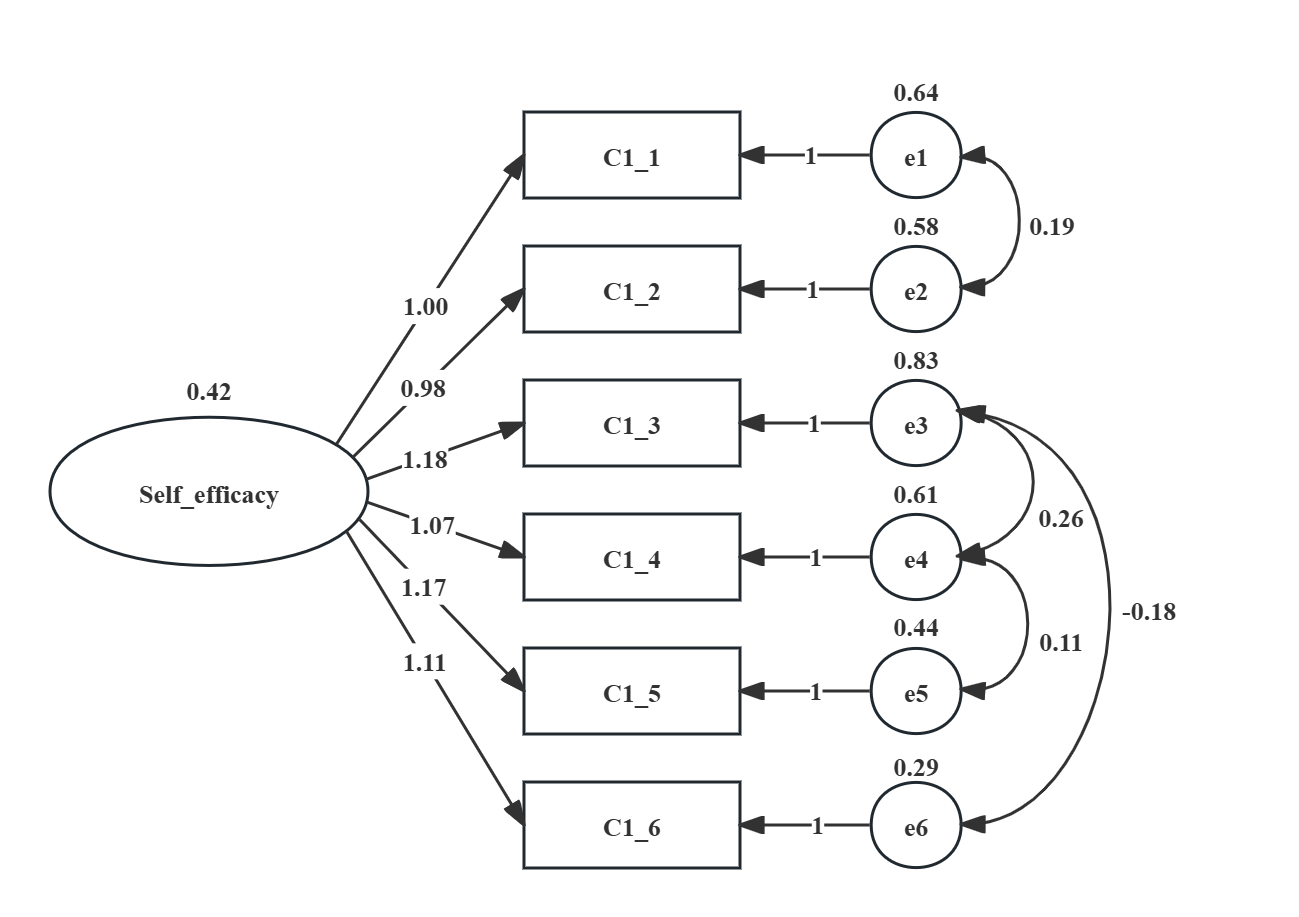

Supplement: Multimedia Appendix 3 [file publichealth-v11-e68400-s003.png]
